# Mitochondrial Dysfunction in PCOS: Insights into Reproductive Organ Pathophysiology

**DOI:** 10.3390/ijms241713123

**Published:** 2023-08-23

**Authors:** Kyle M. Siemers, Abigail K. Klein, Michelle L. Baack

**Affiliations:** 1 Physician Scientist (MD/Ph.D.) Program, Sanford School of Medicine, University of South Dakota, 414 E. Clark Street, Vermillion, SD 57069, USA; kyle.siemers@coyotes.usd.edu; 2 Division of Basic Biomedical Sciences, Sanford School of Medicine, University of South Dakota, Lee Medical Building, 414 E. Clark St., Sioux Falls, SD 57069, USA; abigail.klein@coyotes.usd.edu; 3 Department of Pediatrics, Division of Neonatology, Sanford School of Medicine, University of South Dakota, 1400 W. 22nd St., Sioux Falls, SD 57105, USA; 4 Environmental Influences on Health and Disease Group, Sanford Research, 2301 E. 60th St., Sioux Falls, SD 57104, USA

**Keywords:** polycystic ovary syndrome, PCOS, ovary, uterus, placenta, mitochondria, biogenesis, mtDNA, metabolism, mitochondrial dynamics, oxidative stress

## Abstract

Polycystic ovary syndrome (PCOS) is a complex, but relatively common endocrine disorder associated with chronic anovulation, hyperandrogenism, and micro-polycystic ovaries. In addition to reduced fertility, people with PCOS have a higher risk of obesity, insulin resistance, and metabolic disease, all comorbidities that are associated with mitochondrial dysfunction. This review summarizes human and animal data that report mitochondrial dysfunction and metabolic dysregulation in PCOS to better understand how mitochondria impact reproductive organ pathophysiology. This in-depth review considers all the elements regulating mitochondrial quantity and quality, from mitochondrial biogenesis under the transcriptional regulation of both the nuclear and mitochondrial genome to the ultrastructural and functional complexes that regulate cellular metabolism and reactive oxygen species production, as well as the dynamics that regulate subcellular interactions that are key to mitochondrial quality control. When any of these mitochondrial functions are disrupted, the energetic equilibrium within the cell changes, cell processes can fail, and cell death can occur. If this process is ongoing, it affects tissue and organ function, causing disease. The objective of this review is to consolidate and classify a broad number of PCOS studies to understand how various mitochondrial processes impact reproductive organs, including the ovary (oocytes and granulosa cells), uterus, placenta, and circulation, causing reproductive pathophysiology. A secondary objective is to uncover the potential role of mitochondria in the transgenerational transmission of PCOS and metabolic disorders.

## 1. Introduction

Polycystic ovary syndrome (PCOS) is a complex disease resulting from a variety of reproductive, endocrine, and metabolic abnormalities [1]. The prevalence of PCOS is between 4 and 18% worldwide [2,3], and it affects as many as 5 million people in the US [4]. The Rotterdam criteria are widely used to clinically diagnose PCOS [5]. Using this tool, patients are required to meet at least two of the three criteria, which include oligo-anovulation, hyperandrogenism, and the presence of polycystic ovaries via ultrasound [6,7,8]. Based on which characteristics are present, PCOS can be further categorized into four subtypes, although the naming of these subtypes varies within the literature. The classical subtype includes all three Rotterdam criteria, whereas the other subtypes are characterized by variations of the diagnostic criteria [7,9,10]. These subtypes are helpful diagnostic tools but have not been associated with specific outcomes [9]. Importantly, many cases of PCOS go undiagnosed or diagnosis is significantly delayed, an indicator that there is a much higher prevalence than reported [9].

The lifestyle management of PCOS focuses on weight management, regular testing of glycated hemoglobin A1c (HbA1c), first- and third-trimester oral glucose tolerance testing during pregnancy, and preventing comorbidities through screening for hypertension, dyslipidemia, and psychological disorders [11]. The recommendation for the medical management of PCOS, alongside lifestyle modifications for both adults and adolescents, includes combined oral contraceptives for irregular cycles, hirsutism, and acne combined with metformin to improve glucose levels and adiposity [12]. Metformin’s low cost and availability have set it apart as the first-line treatment for PCOS symptoms; however, many studies are also investigating the efficacy of anti-obesity drugs like glucagon-like peptide-1 agonists as additional therapy [13]. For reproductive outcomes, letrozole has been shown to improve pregnancy rates and live births, as well as reduce time-to-pregnancy [14]. Letrozole may have benefits in combination with metformin, although more studies are needed to better understand the periconceptual and fetal effects of metformin [9].

Key features in the pathophysiology of PCOS are hyperandrogenism due to ovarian androgen hypersecretion [15] and insulin resistance [16]; however, the connections and underlying mechanisms linking these features are not well understood. PCOS has heritable phenotypes but with variability that may be related to maternally inherited mitochondria or epigenetic programming [17,18]. In rodent models, there is even evidence of the transgenerational transmission of PCOS due to the changes in epigenetics and mitochondrial function in oocytes related to dihydrotestosterone (DHT) [19].

**Figure 1:**
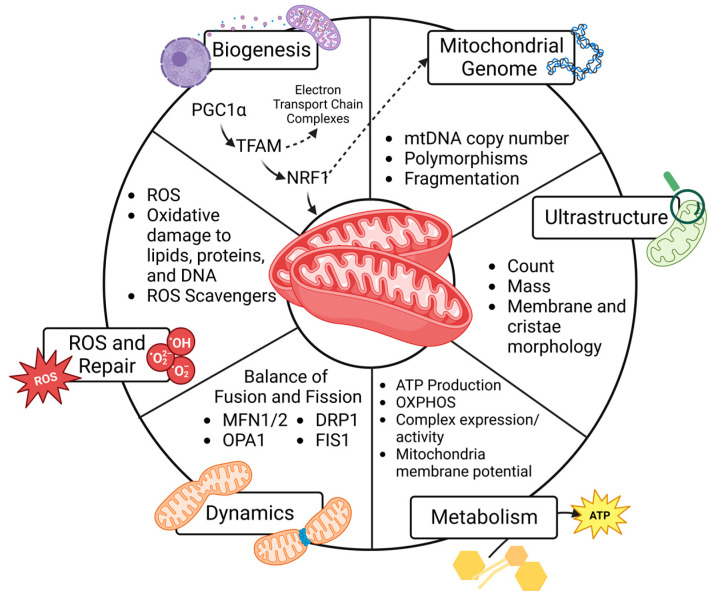
Key elements that regulate mitochondria number and function to influence cell processes and fate. Created with BioRender.com.

**Figure 2:**
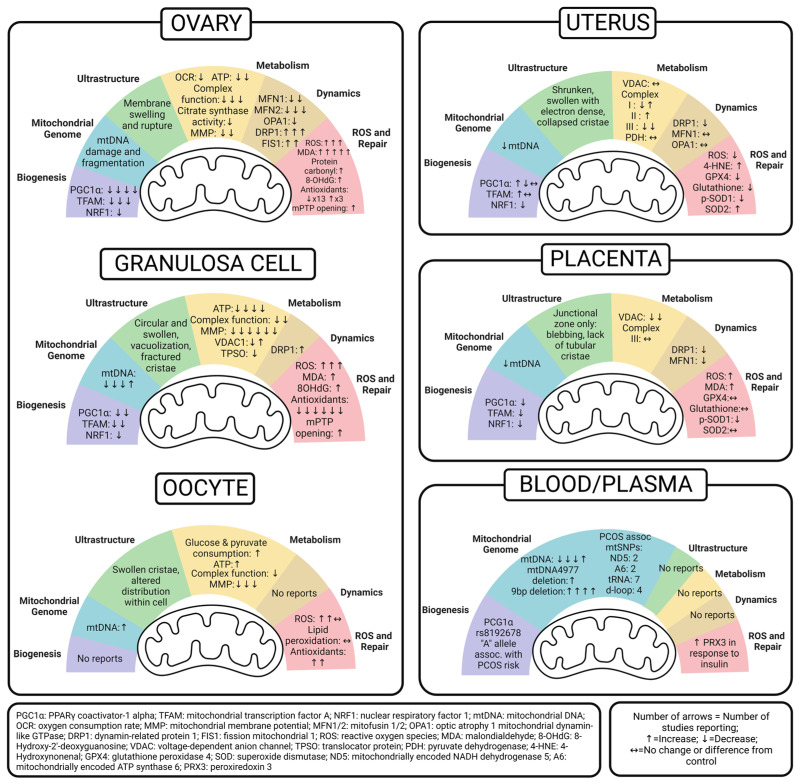
Summary of reported alterations in mitochondrial biogenesis, genome, ultrastructure, metabolism, dynamics, and ROS in the ovary, oocyte, granulosa cell, uterus, placenta, and peripheral blood and plasma of PCOS. Offspring exposure studies were excluded. Created with BioRender.com.

Although the constellation of symptoms associated with PCOS was first documented in 1721 [20,21], the underlying mechanisms remain poorly understood, and the prevalence of PCOS continues to rise. Even with lifestyle changes and medical treatment, patients experience substantial consequences of PCOS across their lifetimes. Moreover, with evidence suggesting that PCOS affects multiple generations, it is crucial to uncover inheritable factors related to the pathogenesis and develop preventative and therapeutic measures to decrease both the reproductive consequences and metabolic co-morbidities in this growing population. Mitochondrial dysfunction has been reported in PCOS, but studies working to uncover the mitochondria-mediated mechanisms of PCOS are limited. The objective of this review is to compile data and examine patterns of mitochondrial dysfunction reported in PCOS to better understand the role of mitochondria in reproductive health and developmental programming. To do this, we review six predefined mitochondrial functions, as shown in Figure 1, and then summarize PCOS-related findings in the ovary (granulosa cells and oocytes), uterus, placenta, and peripheral circulation. The emerging patterns are illustrated in Figure 2. We hope this compilation will set the groundwork needed to develop new diagnostic and therapeutic interventions for people with PCOS and mitigate the transgenerational consequences for their progeny.

## 2. Methods

EvidenceHunt [22] and PubMed were used to search articles published before July 2023 using the search terms “PCOS”, “polycystic ovary syndrome”, “mitochondria”, and/or “antioxidant”. The initial search terms returned 69 articles in PubMed. From there, the authors reviewed these articles for quality and organized them by the relevant organs studied, excluding 21 articles that did not investigate mitochondrial function in the ovary, granulosa cells, uterus, placenta, and peripheral blood and plasma. Three additional studies were excluded because they reported peripheral markers of mitochondrial function in PCOS patients based on calculations of mitochondrial respiration in muscle and plasma MDA levels, which were insufficient to draw reproductive conclusions. The authors reviewed the remaining 45 studies and compiled them into six predefined mitochondria roles in PCOS models, as outlined in Figure 1. Finally, these studies’ results were summarized by organ, as shown in Figure 2, alongside a thorough compilation, as detailed in each section with Table 1, Table 2, Table 3 and Table 4. The outcomes of studies related to measurements of SIRT1/3 and cellular apoptosis that did not also measure predetermined mitochondrial functions were outside the scope of this study. Additionally, studies not examining one of the selected reproductive organs were not included in this review. Because gene symbol capitalization varies between species, unless specifically indicated, this review reports genes and their functional proteins with the protein symbol in all capital letters for consistency across outcomes [23].

## 3. Animal and Cellular Models of PCOS

Animal models are necessary to understand the impact of PCOS on the reproductive system, namely the ovary, uterus, and placenta, as well as inheritance for offspring. The three most common methods of PCOS induction in animal models include dehydroepiandrosterone (DHEA), DHT, or letrozole injections. Both letrozole and DHEA are typically given daily for 20–21 days, whereas DHT administration is more variable, with some studies using daily injections, implantation of controlled-release pellets, or three consecutive injections after the confirmation of pregnancy to investigate in utero exposure. In addition, some studies use these agents in combination with a high-fat diet and/or other methods of inducing insulin resistance, primarily because of the strong association with human PCOS. Interestingly, one study induced PCOS symptoms of anovulation, obesity, and metabolic disorder in a mouse model through a high-fat/high-glucose diet alone [24]. 

Cellular models are also used to understand PCOS pathogenesis. The isolation of primary granulosa cells (GCs), oocytes, and whole organs from PCOS-induced animals is commonly used. Collecting primary tissue like ovaries from humans is not usually feasible; however, PCOS patients undergoing oocyte retrieval in a fertility clinic have both oocytes and GCs collected from the ovaries. This offers an opportunity to study two types of primary cells for the translational study of human disease, albeit in a slightly biased patient population that is seeking fertility treatment. There are also immortalized GC lines, with the most common one in this review being KGN, which is a steroidogenic human ovarian cell line derived from a GC tumor. Some in vitro studies in this review added various concentrations of DHEA and DHT to cell culture systems to mimic androgen exposure. In addition, one study incorporated palmitic acid to recapitulate a high-fat or lipotoxic environment [24]. Overall, these in vitro models are important tools used to understand the role of mitochondria in cellular metabolism, energetic processes, and cell fate.

## 4. Mitochondrial Function and Cell Fate

Both the quantity and quality of mitochondria influence cell function and fate. These vary widely between cell types, primarily because of varying metabolic demands. Mitochondrial biogenesis relies on intact mitochondrial DNA (mtDNA) and robust nuclear and mitochondrial cross-talk to generate new regulatory proteins. The quality is influenced by the ultrastructure of the organelle and the ability to efficiently produce ATP through oxidative phosphorylation (OXPHOS). Dynamics, or fission and fusion, influences mitochondrial quality by facilitating the turnover of damaged mitochondria. As primary producers of reactive oxygen species (ROS), mitochondria rely on cellular antioxidants to prevent oxidative damage to lipids, proteins, and DNA that can set up a vicious cycle of further mitochondrial damage and eventual cell death. These six functions, which are shown in Figure 1 and detailed below, all impact mitochondrial health and disease risk.

### 4.1. Mitochondrial Biogenesis

Mitochondrial biogenesis refers to the collaborative effort between the nucleus and existing mitochondria to generate new proteins that contribute to the cellular network of mitochondria. Biogenesis usually increases in response to rising energetic demands, including proliferation, but it may also increase to replace damaged mitochondria following environmental stressors and/or oxidative stress [25]. For this reason, increased biogenesis may represent a positive or negative cellular response. The generation of new mitochondria through biogenesis is held in balance with mitophagy, the removal of damaged mitochondria. Mitophagy is critical for the quality control of mitochondria, and an imbalance of a high number of dysfunctional mitochondria impairs efficient energy production and creates ROS, which can incite inflammation and signal cell death. 

Biogenesis is under the influence of nuclear and mitochondrial transcriptional regulation. Peroxisome proliferator-activated receptor-gamma coactivator (PGC-1α), the master regulator of mitochondrial biogenesis, resides in the nucleus. Its activation by phosphorylation stimulates a series of transcription factors, including nuclear respiratory factor-1 (NRF-1), NRF-2, estrogen-related receptor-a (ERR-a), and transcription factor A (TFAM). NRF-1 increases the transcription of nuclear-encoded genes that regulate mitochondrial function, and NRF-2 increases the transcription of genes important for redox homeostasis. TFAM is the final transcription factor that begins the transcription and replication of mtDNA [25]. The translation of mtDNA in combination with a variety of nuclear genes generates new mitochondrial preproteins in the cytosol, which are transported into the mitochondrial matrix for sorting and assembly. Because the quantity of mitochondria in cells is not exclusively due to synthesis, it is important to acknowledge that solely measuring the expression of these nuclear transcription factors cannot equate to mitochondrial mass. This is also true because protein activity is also regulated by post-translational modification (phosphorylation). Moreover, increased expression can occur in response to metabolic cues or mitochondrial turnover (mitophagy) related to dysfunctional or damaged mitochondria [26]. It is important to consider the structural and functional aspects that follow the initial transcription of nuclear and mitochondrial genes, protein translation, and mitochondrial localization and assembly to understand the results of biogenesis studies on mitochondrial physiology and pathophysiology. 

### 4.2. Mitochondrial Genome

Measuring the mtDNA copy number can serve as a marker of mitochondrial quantity within a cell, especially when assessed in combination with the imaging and expression of mitochondrial proteins. For this reason, it is often used as a surrogate marker of biogenesis. Importantly, sequencing mtDNA can identify the polymorphisms of 37 genes specific to the mitochondria [27]. Specifically, genetic mutations and heteroplasmy contribute to disease processes. Because mtDNA is maternally inherited, identifying polymorphisms and other mutations in mtDNA may predict disease risk for offspring, especially when considering family history [28,29]. Moreover, mtDNA damage and mutations increase with age and after environmental exposure, so they can be followed longitudinally to assess health and disease risk over time [30,31]. Although mtDNA is important, it should be recognized that mtDNA encodes only 37 genes, and many mitochondrial functions are regulated by proteins encoded by the nuclear genome.

### 4.3. Ultrastructure

The assessment of mitochondrial ultrastructure is also a useful tool for assessing mitochondrial quality. Some studies estimated the integrity and abundance of mitochondria by measuring the expression of the translocase of the outer membrane (TOMM20). This is the most highly expressed protein complex in the outer membrane system [32]. The advent of electron microscopy in the 1950s significantly advanced understanding. It not only allowed for the visualization of mitochondria [33] but also a better understanding of the wide range of structural variations exhibited by different tissues and pathologies [34]. The conformation of the inner and outer membranes that make up the mitochondria’s cristae is crucial for the organelle’s bioenergetic function. An abnormal ultrastructure can lead to ROS production, mitophagy, and ultimately, cellular processes like apoptosis. A multitude of structural abnormalities in cristae have been reported through the use of electron microscopy, many of which are also linked to impaired bioenergetics. Because the inner mitochondrial membrane (IMM) houses the components of the electron transport chain, its surface area and integrity are important for generating the driving force for ATP production [35]. Disease processes such as diabetes are associated with altered cristae structure or a loss of cristae altogether. Although there are other reviews that have explored electron microscopy to uncover complex and crucial protein structures within the inner and outer mitochondrial membranes [36,37], this review aims to leverage studies’ conclusions on mitochondrial ultrastructure to identify patterns that impact metabolism, dynamics, and oxidative stress in PCOS.

### 4.4. Metabolism

There are many ways to assess the role of mitochondria in cellular bioenergetics and ATP production in PCOS. Measuring mRNA or protein expression of the mitochondria-specific protein complexes that participate in OXPHOS, ATP synthase, fatty acid oxidation, and the Krebs cycle can help estimate their abundance. However, it is crucial to also measure these protein complexes’ activities. There are tools to measure the activity of Complexes I–IV of the electron transport chain, as well as citrate synthase and pyruvate dehydrogenase, which are necessary for the Krebs cycle [38,39]. While protein measurements and activity assays are often carried out on mitochondrial isolates, it is important to remember that isolating mitochondria disrupts access to stored fuels such as glycogen and lipid droplets. It also disrupts the cellular mitochondrial network and interactions with other organelles. Therefore, measuring respiration in isolated mitochondria may not fully recapitulate the cell’s metabolic capacity [40]. For this reason, extracellular flux analysis, which measures cellular bioenergetics in intact cells in real time, has advantages [41]. Finally, generating ATP also requires the maintenance of an electrochemical gradient across the IMM. The voltage-dependent anion channel (VDAC) is a protein on the outer mitochondrial membrane (OMM) that facilitates communication between the mitochondria and the rest of the cell; it can serve as a useful marker of the maintenance of the membrane potential [42]. Measuring mitochondria membrane potential (MMP) not only gives insight into the cell’s ability to generate ATP but also its ability to carry out other energy-dependent processes such as the transport of mitochondrial proteins, mitophagy, and ultimately, cell viability [43]. 

### 4.5. Dynamics

Mitochondrial dynamics refers to the fission and fusion events across the networks of mitochondria. The balance of fission and fusion in a cell regulates the number, volume, and position within the cell [44]. Fission may play a role in replication during cell division and marking damaged mitochondria for mitophagy. Fusion to other subcellular organelles, such as lysosomes, is also important for mitochondrial quality control. Therefore, a balance of fission and fusion is necessary for maintaining normal mitochondrial function. An imbalance or pro-fission state is associated with pathophysiological conditions and human disease. For example, under stress, mitochondria become more fragmented, small, and dysfunctional, which makes them more prone to mitophagy [45]. 

More and more human diseases are associated with mutations in proteins governing fission and fusion, so it is important to understand how PCOS influences dynamics in the context of disease risk. The proteins involved in mitochondrial fusion are mitofusins 1 and 2 (MFN1 and MFN2), which drive OMM fusion, and optic atrophy 1 mitochondrial dynamin-like GTPase (OPA1), which facilitates IMM fusion [46]. Mitochondrial fission is primarily carried out by dynamin-related/-like protein 1 (DRP1, also known as DMN1L), which, under the influence of phosphorylation, binds to partners like mitochondrial fission 1 protein (FIS-1) to constrict the organelle [47]. In this review, we report altered dynamics as changes in the abundance or expression of these five proteins or any imaging modalities used to track mitochondria network fusion or fission. 

### 4.6. Reactive Oxygen Species and Repair

Many studies reported oxidative stress in PCOS. Mitochondria are the primary producers of ROS, and ROS generation increases with mitochondrial dysfunction. Although ROS generation is crucial for intracellular redox signaling, excess ROS production causes lipid, protein, and DNA oxidation, which can damage mtDNA and unfolded proteins, leading to a vicious cycle of worsening mitochondrial function, oxidative damage, and cell death [48,49]. Ultimately, an imbalance of ROS production and antioxidant capacity leads to multiple human diseases [50].

There are a variety of ways to measure ROS and downstream oxidative stress, including mitochondrial permeability transition pore (mPTP) opening; direct or indirect measurements of ROS; and lipid, protein, and DNA oxidation. mPTP opening can be measured in a variety of ways. The studies we reviewed utilized the release of mitochondrial cytochrome C [51] and optical density changes following mPTP induction of isolated mitochondria with calcium chloride [52], which are represented by a rate and percent change, respectively. Most commonly, lipid peroxidation is measured using malondialdehyde (MDA) or 4-hydroxynonenal (4-HNE) assays. Protein carbonylation and 8-Hydroxy-2’-deoxyguanosine (8-OHdG) are used to measure protein and DNA oxidation, respectively. Additionally, measuring antioxidant expression and activity can estimate the response to increasing levels of ROS and inform about the cell’s ability to prevent oxidative damage. Common assays include measuring superoxide dismutase (SOD), glutathione peroxidase (GPX), and catalase (CAT). 

On their own, elements of mitochondrial biogenesis, genetics, ultrastructure, metabolism, dynamics, or oxidative stress do not provide a complete understanding of how disease processes impact cell and organ function. Taken together, they can provide a clearer picture of how mitochondrial function in a cyclical and dynamic fashion works to impact disease pathogenesis. Often, the difficulty of utilizing all these modalities simultaneously to assess mitochondrial function limits the conclusions made by studies in isolation. This review investigates the many facets of mitochondrial function in PCOS models to better understand the role of mitochondria and oxidative stress in various reproductive organs. 

## 5. Ovary Mitochondria in PCOS

The ovary is a vital female reproductive endocrine organ, which consists of both somatic cells and germ cells (oocytes) that influence the health of both the parent and the offspring. It is also the organ primarily affected by PCOS. Because of this dual role, we examine the effect of PCOS on ovarian mitochondria as a whole, and we also take a closer look at the granulosa cells (GCs) and oocytes separately. The study methods and results are detailed in Table 1, Table 2 and Table 3. Figure 2 summarizes the emerging patterns of mitochondrial dysfunction in the PCOS ovary to better understand its impact on fertility and generational consequences.

### 5.1. Whole Ovary

On the whole, we found that mitochondrial biogenesis and downstream mitochondrial quantity and quality have been evaluated in a wide variety of PCOS models. Even with diverse methods of PCOS induction, rodent models evaluating biogenesis in whole ovary uniformly show decreased levels of PGC-1α, NRF-1, and TFAM [52,53,54,55]. Metformin treatment increased PGC-1α, NRF-1, and TFAM expression, as did a traditional Chinese medicine, cangfudaotan decoction [52], neurokinin-B antagonist [54], two different carnitine formulations [55], and the overexpression of SIRT3, an NAD-dependent deacetylase [56]. 

Only one study directly evaluated mtDNA in ovarian tissue [52]. Jiang et al. reported more damaged and fragmented mtDNA in the ovaries of a letrozole-induced rat model of PCOS that was also given a high-fat diet, but the administration of either cangfudaotan or metformin significantly decreased mtDNA damage and fragmentation [52]. 

There was little reporting of mitochondria ultrastructure within the ovary as a whole, and most studies examined this within specific cell types like the GCs and oocytes. These are detailed in subsequent sections. Jiang et al. reported mitochondrial membrane swelling and rupture in ovarian tissue and found that cangfudaotan or metformin decreased the total percentage of damaged mitochondria, although not as low as in the control groups [52]. Together Jiang’s findings suggest that mtDNA damage also affects ultrastructure, respiration, and downstream energy production.

Many other studies assessed ATP levels, complex proteins, and citrate synthase in order to better understand the functional role of ovarian mitochondria in PCOS. In general, ATP levels were lower in PCOS-exposed ovaries [52,57], which could be explained by decreased biogenesis and mitochondrial damage. Interestingly, studies of neonatal ovaries exposed to PCOS in utero had ATP levels similar to the controls [58]. Uniformly, all other rodent PCOS studies, excluding the in utero-exposed models, also reported lower Complexes I, III, and IV, as well as total complex enzyme activity [51,52,57]. Although the in utero-exposed model did not exhibit complex enzymatic activity, the authors reported a lower expression of a specific subunit within Complex IV compared to the controls [58]. Impaired complex activity in PCOS ovaries was rescued by cangfudaotan, Bushen Huatan Granules, or selenium delivered by nanoparticles [51,52,57]. One study found that PCOS was associated with lower citrate synthase activity but this increased with the administration of metformin or sodium selenite [59]. Furthermore, two similar studies uniformly reported that PCOS lowers the MMP, which generates ATP-driving gradients in rat ovaries [52,57]. In both cases, cangfudaotan and selenium nanoparticles, along with metformin alone, improved MMP.

Only a couple of studies measured real-time cellular bioenergetics to assess mitochondrial respiration. One study found that PCOS led to a lower oxygen consumption rate (OCR) and respiratory control rate (RCR) in isolated mitochondria [52]. The other study examined bioenergetics through extracellular flux analyses in whole neonatal ovaries from an in utero PCOS-exposed rat model. PCOS-exposed neonatal ovaries had increased basal, maximal, and ATP-linked OCR, along with an increased proton leak [58]. Together, these findings suggest that PCOS impairs mitochondrial respiration in the adult ovary, but exposing the developing ovary to PCOS in utero causes a responsive increase in mitochondrial oxygen consumption that could be associated with oxidative stress across a lifespan. 

In studies evaluating the mitochondrial dynamics in the whole ovary, a common theme of imbalance emerged, specifically with impaired fusion and increased fission, a state that increases mitophagy and oxidative stress while also impairing important networking with other organelles. For fusion, rodent studies found lower levels of MFN1, MFN2, and OPA1, but their expression levels increased with interventions of cangfudaotan, sodium selenite, or metformin [52,53,59]. The fission genes DRP1 and FIS1 were higher in rat PCOS models but decreased with cangfudaotan, sodium selenite, or metformin [52,53,59]. 

Many studies evaluated oxidative stress in PCOS ovaries. Overall, PCOS increased mitochondrial superoxide [51,53], lipid peroxidation (MDA), protein oxidation, and DNA oxidation [53,57,59,60,61]. Cangfudaoton reversed elevated ROS production [52]. Selenium, sodium selenite, metformin, and genistein all successfully decreased MDA levels [53,57,59,60,61], and metformin and sodium selenite decreased protein carbonyls [52,59]. Genistein treatment reversed DNA oxidation in DHEA-induced PCOS [61].

In addition to oxidative damage, some studies evaluated whether PCOS could alter the antioxidant capacity in the ovary, which would significantly influence its ability to respond to oxidative stress. Overall, the studies suggest that PCOS lowers antioxidant capacity, specifically SOD1 mRNA expression and SOD activity, in ovary tissue [53,54,57,59,60,61]. In contrast, two other studies found increased levels of SOD2 protein [53,55], which may be a cellular response to oxidative stress. Others reported impaired GSH, GPx, GR, and GSH:GSSG ratio, whereas one study opposed this with reports of increased GSH-Px [53,57,59,61]. Three different studies found decreased CAT activity, which was rescued with both genistein and neurokinin-B antagonists [54,60,61]. Studying both ROS production and antioxidant capacity is important because when ROS is in excess of antioxidant capacity, oxidative damage and cell death occur. This is highlighted by two studies that also looked at mitochondrial permeability transition pore (mPTP) opening and levels of cytochrome C in the cytosol, both of which are primary regulators of cell death. PCOS increased these markers in rat ovaries, but they were significantly reduced with cangfudaotan and metformin, or Bushen Huatan Granules, respectively [51,52]. We suggest that differences in antioxidant capacity within these studies are related to both the methods of PCOS induction and the timing of tissue collection, as antioxidant capacity may initially be “overwhelmed” by ROS but increase over time in response to ongoing oxidative stress.

**Table 1:** Effects of PCOS on mitochondrial function in the ovary.

Mitochondrial Function	Effect of PCOS Condition on Mitochondrial Function	Model—Species	PCOS Model/Diagnosis	Treatment Timeframe	Method	Therapeutic Intervention	Reference
**Biogenesis**	Decreased PGC1α	Rat	IP Letrozole + HFD	21 days, 21 days, 35 days, 12 weeks	qPCR, WB	Cangfudaotan (IG) and metformin (IG) increased PGC1α to control levels	[52]
		Rat	IG Letrozole	21 days	WB		[53]
		Mouse	SQ DHT	35 days	WB	Overexpressing SIRT3 in vivo increased PGC1α back to control levels	[56]
		Mouse	HF/HGD (58% kcal fat + sucrose)	12 weeks	qPCR	Neurokinin-B antagonist increased PGC1α back to control levels	[54]
	Decreased TFAM	Mouse	SQ DHEA	20 days	WB	(1) L-carnitine (LC) + acetyl-L-carnitine (ACL) (2) LC and ACL plus propionyl-L-carnitine Both formulations increased TFAM compared to DHEA alone and controls	[55]
		Rat	IG Letrozole	21 days	WB		[53]
		Mouse	HF/HGD (58% kcal fat + sucrose)	12 weeks	qPCR	Neurokinin-B antagonist increased TFAM back to control levels	[54]
	Decreased NRF1	Mouse	HF/HGD (58% kcal fat + sucrose)	12 weeks	qPCR	Neurokinin-B antagonist increased NRF1 back to control levels	[54]
**Mitochondrial Genome**	Increased mtDNA fragmentation	Rat	On day 22 of HFD (46% fat), OG letrozole	21 days	qPCR	Cangfudaotan (IG) and metformin (IG) decreased mtDNA damage and fragmentation	[52]
**Ultrastructure**	Membrane swelling and ruptures	Rat	IP Letrozole + HFD	21 days	EM	% of total damaged mitochondria decreased with either metformin (IG) or cangfudaotan (IG) but were still higher than control levels	[52]
**Metabolism**	Increased basal, maximal and ATP-linked OCR, proton leak	Mice—offspring	DHT injection in dams post-coitus, assessed pup neonatal ovaries	GD 16.5, 17.5, 18.5	XF (Agilent) of whole neonatal ovaries		[58]
	Decreased OCR, RCR	Rat	IP letrozole + HFD	21 days	Oxytherm Clark-type electrode on isolated mitochondria	Cangfudaotan (IG) increased OCR, RCR	[52]
	Decreased ATP	Rat	On day 22 of HFD (46% fat), OG letrozole	21 days	Colorimetric ATP assay	SeNP alone and in combination with metformin increased ATP (most increase in combination)	[57]
		Rat	IP letrozole + HFD	21 days	ATP assay	Cangfudaotan (IG) increased ATP levels	[52]
	No difference in ATP	Mice—offspring	DHT injection in dams post-coitus, assessed pup neonatal ovaries	GD 16.5, 17.5, 18.5	XF (Agilent) of whole neonatal ovaries		[58]
	Decreased activity of mitochondrial complex enzymes	Rat	IP letrozole + HFD	21 days	Complex enzyme activity assays	Cangfudaotan (IG) increased mitochondrial complex activity	[52]
	Decreased Complex I activity	Rat	On day 22 of HFD (46% fat), OG letrozole	21 days	Complex I enzyme activity assay	SeNP alone and in combination with metformin increased Complex 1 activity (most increase in combination)	[57]
		Rat	SQ DHEA	20 days		Bushen Huatan Granules (OG) increased activity of Complex I	[51]
	Decreased Complexes III, IV activity		SQ DHEA	20 days	Complexes III, IV enzyme activity assays	Bushen Huatan Granules (OG) increased activity of Complexes III and IV	[51]
	Decreased Complex IV (Cox6a2 subunit)	Mice—offspring	DHT injection in dams post-coitus, assessed pup neonatal ovaries	GD 16.5, 17.5, 18.5	RNAseq		[58]
	Decreased citrate synthase activity	Rat	OG letrozole	21 days	Citrate synthase activity assay	Metformin (OG) and sodium selenite (OG) increased mitochondrial citrate synthase activity but still lower than control group	[59]
	Decreased MMP	Rat	On day 22 of HFD (46% fat), OG letrozole	21 days	JC-1 staining	SeNP alone and in combination with metformin increased MMP (most increase in combination)	[57]
		Rat	IP letrozole + HFD			Cangfudaotan (IG) or metformin (IG) increased MMP	[52]
**Dynamics**	Decreased MFN1	Rat	IP letrozole + HFD	21 days	qPCR/WB	Cangfudaotan (IG) or metformin (IG) increased MFN1	[52]
		Rat	IG Letrozole	21 days	WB		[53]
	Decreased MFN2	Rat	IP letrozole + HFD	21 days	qPCR/WB	Cangfudaotan (IG) or metformin (IG) increased MFN2	[52]
		Rat	OG letrozole (OG)	21 days	qPCR/ELISA kit	Metformin (OG) and sodium selenite (OG) increased MFN2 but still lower than control group	[59]
		Rat	IG Letrozole	21 days	WB		[53]
	Decreased OPA1	Rat	IP letrozole + HFD	21 days	qPCR/WB	Cangfudaotan (IG) or metformin (IG) increased OPA1	[52]
	Increased DRP1	Rat	IP letrozole + HFD	21 days	qPCR/WB	Cangfudaotan (IG) or metformin (IG) decreased DRP1	[52]
		Rat	OG letrozole	21 days	qPCR/ELISA kit	Metformin (OG) and sodium selenite (OG) decreased DRP1 but still higher than control group	[59]
		Rat	IG Letrozole	21 days	WB		[53]
	Increased FIS1	Rat	IP letrozole + HFD	21 days	qPCR/WB	Cangfudaotan (IG) or metformin (IG) decreased FIS1	[52]
		Rat	IG Letrozole	21 days	WB		[53]
**ROS and Repair**	Increased ROS	Rat	IP letrozole + HFD	21 days	DCF staining	Cangfudaotan (IG) or metformin (IG) decreased ROS	[52]
		Rat	IG Letrozole	21 days	Activity to produce superoxide anion assay		[53]
	Increased mitochondrial superoxide	Rat	SQ DHEA	20 days	MitoSOX staining	Bushen Huatan Granules (OG) decreased mitochondrial superoxide	[51]
	Increased lipid peroxidation	Rat	On day 22 of HFD (46% fat), OG letrozole	21 days	MDA assay	SeNP alone or in combination with metformin decreased lipid peroxidation	[57]
		Rat	OG letrozole	21 days		Metformin (OG) and sodium selenite (OG) decreased lipid peroxidation but still higher than control group	[59]
		Rat	SQ DHEA	21 days			[60]
		Rat	IG Letrozole	21 days			[53]
		Mouse	SQ DHEA	20 days		Genistein decreased lipid peroxidation	[61]
	Increased protein oxidation	Rat	OG letrozole	21 days	DNPH reaction assay	Metformin (OG) and sodium selenite (OG) decreased protein oxidation but still higher than control group	[59]
	Increased DNA oxidation	Mouse	SQ DHEA	20 days	8-OHdG ELISA	Genistein decreased DNA oxidation levels	[61]
	Decreased antioxidant capacity	Rat	OG letrozole	21 days	Ferric-reducing antioxidant power assay	Metformin (OG) and sodium selenite (OG) increased antioxidant capacity but still lower than control group	[59]
	Decreased SOD activity	Rat	On day 22 of HFD (46% fat), OG letrozole	21 days	SOD enzyme activity assay	SeNP alone or in combination with metformin increased SOD levels	[57]
		Rat	SQ DHEA	21 days			[60]
		Mouse	SQ DHEA	20 days		Genistein increased SOD	[61]
	Decreased SOD1	Mouse	HF/HGD (58% kcal fat + sucrose)	12 weeks	qPCR	Neurokinin-B antagonist increased SOD1	[54]
	Increased SOD2 (MnSOD)	Mouse	SQ DHEA	20 days	WB	(1) LC + ACL and (2) LC, ACL + propionyl-L-carnitine both decreased SOD2	[55]
		Rat	IG Letrozole	21 days	WB		[53]
	Decreased GSH	Rat	On day 22 of HFD (46% fat), OG letrozole	21 days	GSH level	SeNP alone or in combination with metformin increased GSH levels	[57]
	Decreased GSH-Px (GPx)	Rat	OG letrozole	21 days	GPx enzyme activity assay	Metformin (OG) and sodium selenite (OG) increased GPx activity but still lower than control group	[59]
		Mouse	SQ DHEA	20 days	GSH-Px level	Genistein increased GSH-Px	[61]
	Increased GSH-Px	Rat	IG Letrozole	21 days	GSH-Px enzyme activity assay		[53]
	Decreased GR	Mouse	SQ DHEA	20 days	GR enzyme activity assay		[61]
	Decreased GSH:GSSG ratio	Mouse	SQ DHEA	20 days	GSH and GSSG levels	Genistein increased GSH:GSSG ratio	[61]
	Decreased CAT activity	Mouse	SQ DHEA	20 days	CAT enzyme activity assay	Genistein increased CAT activity	[61]
		Rat	SQ DHEA	21 days			[60]
		Mouse	HF/HGD (58% kcal fat + sucrose)	12 weeks	qPCR	Neurokinin-B antagonist increased CAT expression	[54]
	Increased opening of mPTP	Rat	IP letrozole + HFD	21 days	Mitochondrial Membrane Pore-Channel Colorimetric Assay	Canfudaton (IG) or metformin (IG) decreased opening of mPTP	[52]
	Increased levels of Cytochrome C in cytosol than in mitochondria	Rat	SQ DHEA	20 days	WB	Bushen Huatan Granules (OG) decreased levels of Cytochrome C in cytosol fraction compared to mitochondrial fraction	[51]

Footnotes for Table 1, Table 2 and Table 3: GC: granulosa cell; PGC1α: peroxisome proliferator-activated receptor gamma coactivator 1-alpha; TFAM: mitochondrial transcription factor A; NRF1: nuclear respiratory factor 1; OCR: oxygen consumption rate; RCR: respiratory control ratio; MMP: mitochondrial membrane potential; MFN1: mitofusin 1; MFN2: mitofusin 2; OPA1: optic atrophy 1 mitochondrial dynamin-like GTPase; DRP1: dynamin-related protein 1; FIS1: fission 1; SOD: superoxide dismutase; SOD1: superoxide dismutase 1; SOD2: superoxide dismutase 2; MnSOD: manganese superoxide dismutase; GSH: reduced glutathione; GSH-Px: glutathione peroxidase; GPx: glutathione peroxidase; GR: glutathione reductase; GSSG: oxidized glutathione; CAT: catalase; mPTP: mitochondrial permeability transition pore; NDUFB8: NADH:Ubiquinone Oxidoreductase Subunit B8; ATP5j: ATP Synthase Peripheral Stalk Subunit F6; VDAC1: voltage-dependent anion-selective channel 1; TSPO: translocator protein; UPR-MT: mitochondrial unfolded protein response; ND1: NADH dehydrogenase 1; ND2: NADH dehydrogenase 2; ND5: NADH dehydrogenase 5; ND6: NADH dehydrogenase 6; CO1: cytochrome c oxidase subunit 1; CO2: cytochrome c oxidase subunit 2; CO3: cytochrome c oxidase subunit 3; IP: intraperitoneal; HFD: high-fat diet; IG: intragastric; SQ: subcutaneous; DHT: dihydrotestosterone; HGD: high-glucose diet; kcal: kilocalories; DHEA: dehydroepiandrosterone; OG: oral gavage; EV: extracellular vesicle; GD: gestational day; EM: electron microscopy; TEM: transmission electron microscopy; XF: extracellular flux analysis; JC-1: mitochondrial membrane potential probe; DCF: Dichlorofluorescein; DCHF-DA: 2′-7′-Dichlorodihydrofluorescein diacetate; MDA: malondialdehyde; BAT: brown adipose tissue; TMRE: Tetramethylrhodamine, ethyl ester; ICC: immunocytochemistry; SIRT3: sirtuin 3; SeNP: selenium nanoparticle; 8-OHdG: 8-hydroxy-2′-deoxyguanosine; si-NK3R: small interfering RNA targeting human NK3R; eCG: equine chorionic gonadotropin.

### 5.2. Granulosa Cell

Similar to the ovary, PCOS-exposed GC studies reported lower levels of the key biogenesis transcription factors, PGC-1α, NRF-1, and TFAM [54,56,62]. Treating GCs with either vitamin D3, small interfering RNA targeting human neurokinin 3 (si-NK3R), or overexpressing SIRT3 increased these transcription factors [54,56,62].

Upon investigation of the mitochondrial genome in GCs, three studies (a mouse, rat, and human model) reported lower mtDNA copy numbers, while one study reported higher mtDNA copy numbers in a KGN cell line incubated with DHT [24,56,62,63]. Vitamin D3 rescued a low mtDNA copy number in a mouse PCOS model [62], whereas the overexpression of SIRT3 lowered the high mtDNA copy number in the KGN PCOS model [56].

Many GC studies characterized the ultrastructure of mitochondria, primarily through transmission electron microscopy within ovarian tissue. Although each study had unique findings related to the ultrastructure, the overall conclusion was that PCOS is associated with abnormal mitochondria ultrastructure. Specific findings included disorganized and fractured cristae, cristae dissolution, mitochondria that were less electron-dense, swollen, less rod-shaped, more circular and constricted, or that had membrane defects, aggregated distribution, and vacuolization [24,58,64,65]. Interestingly, some of these ultrastructural abnormalities were also seen in neonatal ovaries exposed to PCOS in utero [58]. Melatonin or equine chorionic gonadotropin rescued some of these abnormalities [64,65]. In addition to electron microscopy, one study used nonyl-acridine orange analyzed by flow cytometry to evaluate the number of mitochondria, and pLV-mitoDsRed plasmid transfection, which tags ATP synthase complex, to evaluate mitochondria by microscopy. Using these techniques, Sreerangaraja et al. reported an overall decrease in the number of mitochondria and mitochondrial mass, along with an increase in fragmentation and constrained cell expansion in human GCs from PCOS patients [66]. Together, these data suggest that both mitochondrial quantity and quality are lower in PCOS GCs.

Overall, PCOS also lowered oxidative capacity and energy production in GCs. Four groups, including rodent, human, and KGN cells, reported lower ATP levels [24,56,61,63]. Six studies reported MMP to be decreased, with representation from each type of model species [56,61,63,64,66]. Treatment with genistein increased ATP and MMP, but treatment with an *Nrf2* inhibitor had less effect on ATP and MMP rescue compared to the genistein group, although they were still higher than in the PCOS group [61]. In addition, overexpressing SIRT3 in GCs increased ATP and MMP, but the addition of a PGC-1α inhibitor reversed these effects [56], which suggests that the number of mitochondria played a role. Overall, PCOS also decreased complex activity (Complexes I, III, and IV), including mRNA and protein levels of NDUFB8, a Complex I subunit, and ATP5j, a subunit of ATP synthase [24,51]. Incubating GCs with serum from PCOS rats treated with Bushen Huatan Granules improved the activities of Complexes I, III, and IV [51]. The only discrepancies in metabolic outcomes came from two conflicting reports on VDAC1. One study, using a rat PCOS model, reported higher VDAC1 protein levels, whereas another study, using GCs from PCOS patients, reported lower *VDAC* mRNA levels and immunocytochemical staining [53,67]. The differences may be due to species differences or gene and protein expression differences.

Only one study evaluated proteins regulating mitochondrial dynamics in GCs. Salehi et al. used a rat PCOS model to show that DRP1 fission protein levels were higher in primary isolated GCs [65]. This is consistent with findings for the whole ovary.

Numerous methods were used to assess oxidative stress in both animal and human PCOS studies. Like the ovary, one mouse model and one human study found that PCOS increased ROS levels in GCs, and genistein decreased ROS in the mouse model [61,63]. They also reported more lipid and DNA oxidative damage that was rescued with genistein [61]. One study reported an upregulation of mitochondrial unfolded protein response (UPR-MT) proteins, which is likely in response to an increase in the amount of damaged or misfolded proteins due to oxidative stress [68]. When examining antioxidant capacity, most studies reported decreased SOD, SOD1, GSH-Px, GR, GSH:GSSG ratio, and CAT in PCOS models, and some studies showed improvement with either genistein or si-NK3R [54,61]. Like the whole ovary, PCOS increased the opening of the mPTP in KGN cells and melatonin reversed this [64]. Overall, our summary shows that PCOS appears to affect mitochondria in GCs similarly to those in the whole ovary.

**Table 2:** Effects of PCOS on mitochondrial function in granulosa cells.

Mitochondrial Function	Effect of PCOS on Mitochondrial Function	Model—Species	PCOS Model/Diagnosis	Treatment Timeframe	Method	Therapeutic Intervention	Reference
**Biogenesis**	Decreased PGC1α	Cell line KGN	500 mM DHT	24 h	WB	Overexpression of SIRT3 (cell transfection) increased PGC1α levels comparable to controls	[56]
		Cell line KGN	Palmitic Acid + DHT	Various	qPCR	si-NK3R increased PGC1α	[54]
	Decreased TFAM	Mouse	SQ DHEA	20 days prior to puberty	qPCR	Vitamin D3 (100 mM for 24 h) increased TFAM	[62]
		Cell line KGN	Palmitic Acid + DHT	Various	qPCR	si-NK3R increased TFAM	[54]
	Decreased NRF1	Cell line KGN	Palmitic Acid + DHT	Various	qPCR	si-NK3R increased NRF1	[54]
**Mitochondrial Genome**	Decreased mtDNA copy number	Mouse	SQ DHT	20 days prior to puberty	qPCR	Vitamin D3 (100 mM for 24 h) increased mtDNA copy number	[62]
		Rat	SQ DHEA	20 days	qPCR (mtND1:beta-globin)		[24]
		Human	Rotterdam		qPCR (mtND1:beta-actin)		[63]
	Increased mtDNA copy number	Cell line KGN	500 nM DHT	24 h	qPCR	Overexpression of SIRT3 decreased mtDNA copy number, which increased back to DHT-exposed levels with PGC1a inhibitor	[56]
**Ultrastructure**	Disorganized cristae, vacuoles, less electron-dense	Mouse—offspring	DHT injection post-coitus, assessed pup neonatal GCs	GD 16.5, 17.5, 18.5	TEM		[58]
	Mitochondrial aggregated distribution, cristae dissolution and fracture, presence of vacuoles (66.66% abnormal mitochondria compared to 0% in controls)	Rat	SQ DHEA	20 days	TEM		[24]
	Mitochondrial swelling and membrane defects	Mouse	SQ DHT	35 days	TEM	Melatonin reduced mitochondrial swelling and membrane defects	[64]
	Mitochondrial swelling and membrane defects	Human	Rotterdam		TEM		[64]
	Less rod-shaped mitochondria, more circular/constricted mitochondria	Rat	Continuous-release DHT pellet implant	1 month (83 μg/day)	TEM	eCG increased rod-shaped mitochondria and decreased circular/constricted mitochondria	[65]
	Decreased number of mitochondria	Human	Rotterdam		Nonyl acridine orange (NAC) flow cytometry		[66]
	Decreased mitochondrial mass, increased mitochondrial fragmentation, constrained cell expansion	Human	Rotterdam		pLV-mitoDsRed plasmid transfection (tags ATP synthase) and imaging		[66]
**Metabolism**	Decreased ATP levels	Mouse	100 μM DHEA	12 h	ATP assay	Genistein increased ATP levels, but still lower than controls. Additional treatment with NRF2 inhibitor ML385 decreased ATP levels compared to genistein alone but still higher than DHEA group	[61]
		Rat	SQ DHEA	20 days			[24]
		Cell line KGN	500 nM DHT	24 h		Overexpression of SIRT3 increased ATP but addition of PGC1a inhibitor reversed this effect	[56]
		Human	Rotterdam				[63]
	Decreased activity of Complexes I, III, IV	Rat	Testosterone (10^−5^ M)	24 h	Complex I/III/IV activity assay	6 h incubation with serum from rats receiving Bushen Huatan Granules treatment led to increased activity of Complexes I, III, IV	[51]
	Decreased NDUFB8 (Complex I subunit) and ATP5j (ATP synthase subunit)	Rat	SQ DHEA	20 days	qPCR/WB		[24]
	Decreased MMP	Mouse	SQ DHEA	20 days	JC-1 flow cytometry	Genistein increased MMP but still lower than controls. Additional treatment with NRF2 inhibitor ML385 had similar MMP to DHEA group	[61]
		Cell line KGN	500 nM DHT	24 h	JC-1 staining	Overexpression of SIRT3 increased MMP but addition of PGC1a inhibitor reversed this effect	[56]
		Cell line KGN	500 nM DHT	24 h		Melatonin (1000 pM for 24 h) increased MMP	[64]
		Human	Rotterdam		TMRE flow cytometry		[66]
		Human	Rotterdam		Mitotracker Red flow cytometry		[66]
		Human	Rotterdam		JC-1 Mitochondrial Membrane Potential Kit		[63]
	Increased VDAC1	Rat	IG letrozole	21 days	WB		[53]
	Decreased VDAC1	Human	Rotterdam		qPCR/ICC		[67]
	Decreased TSPO	Human	Rotterdam		qPCR/ICC		[67]
**Dynamics**	Increased DRP1	Rat	Continuous-release DHT pellet implant	1 month (83 μg/day)	WB		[65]
**ROS and Repair**	Increased ROS	Mouse	100 μM DHEA	12 h	DCFH-DA flow cytometry	Genistein decreased ROS	[61]
		Human	Rotterdam		DCFH-DA		[63]
	Increased mitochondrial superoxide	Cell line KGN	500 nM DHT	24 h	MitoSOX staining	Overexpression of SIRT3 decreased mitochondrial superoxide but addition of PGC1-a inhibitor reversed this outcome	[56]
	Increased lipid peroxidation	Mouse	SQ DHEA	20 days	MDA assay	Genistein decreased lipid peroxidation	[61]
	Increased DNA oxidation	Mouse	SQ DHEA	20 days	8-OHdG ELISA	Genistein decreased 8-OhdG levels	[61]
	Decreased SOD, GSH-Px, GR, GSH:GSSG ratio	Mouse	SQ DHEA	20 days	SOD, GR enzyme activity assays/GSH-Px, GSH:GSSG content assays	Genistein increased SOD, CAT, GSH-Px, GSH:GSSG ratio	[61]
	Decreased SOD1	Cell line KGN	Palmitic Acid + DHT	Various	qPCR	si-NK3R increased SOD1	[54]
	Decreased CAT	Mouse	SQ DHEA	20 days	CAT enzyme activity assay	Genistein increased CAT	[61]
		Cell line KGN	Palmitic Acid + DHT	Various	qPCR	si-NK3R increased CAT	[54]
	Upregulation of UPR-MT (mitochondrial unfolded protein response) proteins	Human	Rotterdam		qPCR		[68]
	Increased opening of mPTP	Cell line KGN	500 nM DHT	24 h	Mitochondrial permeability transition pore assay	Melatonin (1000 pM for 24 h) decreased opening of mPTP	[64]

### 5.3. Oocyte

Evaluating the oocytes in PCOS models provides a unique opportunity to understand the pathogenesis of infertility, as well as the consequences for the preimplantation of embryos and generational disease risk. Both mitochondrial quantity and quality are important for oocyte development and maturation. Because mitochondria are maternally inherited, passing on dysfunctional or damaged mitochondria can lead to detrimental lifelong health outcomes in the developing offspring. The very earliest stage of oocyte development, the primordial germ cell, contains the lowest number of mitochondria but undergoes significant amplification during oocyte maturation so that the mature oocyte contains about 100,000 mitochondria. This high number of mitochondria is essential for fertilization, proliferation, and implantation, which are all high-energy events. 

In studies evaluating oocytes exposed to PCOS, there were no reports that focused on mitochondrial biogenesis, but two studies assessed the mitochondrial genome. The first study collected oocytes from a mouse model of PCOS and, unlike in the whole ovary, they found an increased mtDNA copy number [69]. Interestingly, a study that focused on multigenerational outcomes found no differences in the mtDNA copy number in oocytes from post-pubertal female rats exposed to PCOS in utero [69,70]. These studies suggest a variable effect of PCOS on the ovary and oocytes, possibly related to protective mechanisms such as heteroplasmy and enhanced DNA repair in the oocytes.

Multiple studies assessed the ultrastructure of oocyte mitochondria, which are unique in that they normally have a more circular appearance and less complex cristae due to their naïve state [71]. Despite this relatively less complex phenotype, some studies found that PCOS caused disorganized, malformed, and swollen cristae; less or no electron-dense contents; and vacuolization [58,69,70]. One interesting study also showed an abnormal mitochondrial distribution in murine oocytes that had been co-cultured with extracellular vesicles from patients with the non-hyperandrogenic phenotype of PCOS according to Rotterdam criteria [72]. 

Alterations in cellular metabolism were also reported in PCOS oocytes. One human study used an ultra-microfluorometric assay to show that PCOS caused increased glucose and pyruvate consumption in oocytes [73]. Multiple studies showed a decrease in MMP in rodent models of PCOS, which was found to be increased in a mouse model of a PCOS study with a rat-to-mouse brown adipose tissue xenotransplant [69,70,74]. Studies reporting ATP levels and complex protein expression were vastly different. Oocytes collected from a PCOS mouse model had higher ATP levels despite lower mitochondrial Complex I gene expression (*Nd1*, *Nd2*, and *Nd5*) [69]. Conversely, young adult offspring exposed to PCOS in utero had no changes in oocyte ATP levels despite increased mitochondrial Complexes I and IV gene expressions (*Nd6* and *Co1*, *Co2*, *Co3*, respectively) [70]. 

We found no studies that reported mitochondrial dynamics in PCOS-exposed oocytes, but multiple studies evaluated oxidative stress. Overall ROS production was increased in three studies, one of which showed an improvement when rat-to-mouse brown adipose tissue xenotransplant was used [74]. Only one study reported no differences in ROS or lipid peroxidation [69]. Oocytes co-incubated with extracellular vesicles from PCOS patients had an increase in both CAT and GSS [72]. When taken together, PCOS is associated with increased ROS and a likely responsive increase in antioxidants in exposed oocytes. Overall, these studies support our previous claim that PCOS has a different effect on adult and developing oocytes from exposed offspring. Although most data show that developing oocytes in exposed offspring have mitochondrial quantity and quality that puts them at risk for oxidative stress across the lifespan, more studies are needed to determine the transgenerational consequences on reproduction and health.

**Table 3:** Effects of PCOS on mitochondrial function in oocytes.

Mitochondrial Function	Effect of PCOS on Mitochondrial Function	Model—Species	PCOS Model/Diagnosis	Treatment Timeframe	Method	Therapeutic Intervention	Reference
**Biogenesis**	No Reports						
**Mitochondrial Genome**	Increased mtDNA copy number	Mouse	Controlled-release DHT pellet implant	90 days (2.75 μg/day)	qPCR (mtCO1:tubulin)		[69]
	No difference in mtDNA copy number	Mouse—offspring	DHT injection in dam post-coitus, assessed post-pubertal pup oocytes	GD 16.5, 17.5, 18.5	qPCR (mtCO1:tubulin)		[58]
**Ultrastructure**	Disorganized cristae, vacuoles, less electron-dense	Mouse—offspring	DHT injection in dam post-coitus, assessed post-pubertal pup oocytes	GD 16.5, 17.5, 18.5	TEM		[58]
	Mitochondria with malformed cristae with concentric circles, swollen or loss of cristae	Mouse	Controlled-release DHT pellet implant	90 days (2.75 μg/day)	SEM		[69]
	Mitochondria have swollen cristae, no electron-dense contents, and are vacuolated	Mouse—offspring	DHT injection in dams post-coitus, assessed post-pubertal pup oocytes	GD 16.5, 17.5, 18.5	TEM		[70]
	Abnormal mitochondria distribution	Human/Mouse	EVs isolated from PCOS patients with non-hyperandrogenic phenotype were co-cultured with control murine oocytes		Mitochondrial Red Fluorescent Probe		[72]
**Metabolism**	Increased glucose, pyruvate consumption	Human	Rotterdam		Ultra-microfluorometric assay		[73]
	Increased ATP levels	Mice	Controlled-release DHT pellet implant	90 days (2.75 μg/day)	ATP assay		[69]
	No difference in ATP levels	Mouse—offspring	DHT injection in dams post-coitus, assessed post-pubertal pup oocytes	GD 16.5, 17.5, 18.5			[70]
	Decreased mitochondrial Complex I genes (ND1, ND2, ND5)	Mice	Controlled-release DHT pellet implant	90 days (2.75 μg/day)	qPCR		[69]
	Increased mitochondrial Complexes I and IV genes (ND1, ND6 and CO1, CO2, CO3)	Mouse—offspring	DHT injection in dams post-coitus, assessed post-pubertal pup oocytes	GD 16.5, 17.5, 18.5			[70]
	Decreased MMP	Mice	Controlled-release DHT pellet implant	90 days (2.75 μg/day)	JC-1 staining		[69]
		Rat	DHEA injection (interscapular region)	20 days		Rat-to-mouse BAT xenotransplant increased MMP	[74]
		Mice—offspring	DHT injection in dams post-coitus, assessed post-pubertal pup oocytes	GD 16.5, 17.5, 18.5			[70]
**ROS and Repair**	Increased ROS	Rat	DHEA injection (interscapular region)	20 days	ROS assay using DCFH-DA	Rat-to-mouse BAT xenotransplant decreased ROS level	[74]
		Human	EVs isolated from PCOS patients with non-hyperandrogenic phenotype were co-cultured with control murine oocytes		DCHF-DA staining		[72]
		Mice—offspring	DHT injection in dams post-coitus, assessed post-pubertal pup oocytes	GD 16.5, 17.5, 18.5	CellROX staining		[70]
	No differences in ROS	Mice	Controlled-release DHT pellet implant	90 days (2.75 μg/day)			[69]
	No differences in lipid peroxidation	Mice	Controlled-release DHT pellet implant	90 days (2.75 μg/day)	BODIPYC11^®^ 581/591 staining		[69]
	Increased CAT	Human	EVs isolated from PCOS patients with non-hyperandrogenic phenotype were co-cultured with control murine oocytes		qPCR		[72]
	Increased GSS	Human	EVs isolated from PCOS patients with non-hyperandrogenic phenotype were co-cultured with control murine oocytes		qPCR		[72]

## 6. Uterus Mitochondria in PCOS

Despite advances in oocyte retrieval and assisted reproductive therapy, poor uterine receptivity and implantation rates continue to be a barrier facing patients with PCOS [75]. Studies investigating uterine mitochondrial function in humans with PCOS are limited. Multiple rodent studies measured the effect of PCOS on mitochondrial function in the uterus. These studies are summarized in Table 4.

The current literature investigating mitochondrial biogenesis in the PCOS-exposed uterus is limited and presents contrasting results. One human study found that cultured endometrium from patients with PCOS had increased mitochondrial TFAM [76]. Similarly, in non-pregnant mice treated with subcutaneous DHEA, PGC-1α, a transcriptional regulator of biogenesis, was also higher [77]. However, the gravid uterus from rats treated with DHT and insulin had no change in or lower *Pgc1a* expression [78,79]. Counter to the human study, Hu et al. found that these pregnant rats do not have higher *Tfam* expression, and *Nrf1* expression is also lower [79]. Flutamide administered alongside DHT and insulin increased the expression of *Nrf1* in these rats [78]. 

Hu et al. also found that the mtDNA copy number was lower in gravid rats treated with both DHT and insulin, but individual treatment had no effect on the copy number [79]. This was the only study we found that reported the mtDNA copy number in the uterus, and no study reported on mutations or fragmentations. Taken together with biogenesis studies, it is possible that metabolic derangements such as hyperglycemia and hyperlipidemia that are associated with PCOS increase mitochondrial quantity in the non-gravid uterus but may result in a relatively lower mitochondrial quantity in the pregnant uterus.

Multiple studies used transmission electron microscopy to assess the ultrastructure of mitochondria in gravid rat uteruses exposed to DHT and insulin. All studies found ultrastructural abnormalities that included collapsed, poorly formed, or absent cristae and small, swollen mitochondria [78,80,81]. Treatment with flutamide reduced the number of small, swollen mitochondria but disorganized cristae were still present [78]. Treatment with n-acetyl-cysteine (NAC) did not rescue ultrastructural changes associated with PCOS, and interestingly, NAC altered mitochondria structure, even in control rats [81]. This study also measured the abundance of prohibitin I, a chaperone protein important for mitochondrial integrity. They found that DHT and insulin increased prohibitin I in the pregnant rat uterus, which did not change with NAC treatment [81]. 

Four studies examined metabolic differences in PCOS-exposed uteruses. One was a human study and three used a similar DHT and insulin-treated rat uterus. Despite using similar models, the rat studies demonstrated conflicting results. One study found increased Complex I and Complex II protein expression [78], whereas others found decreased Complexes I and III expression [79,81]. High-dose NAC normalized Complex I and III levels in PCOS uteruses but also decreased Complexes I and V abundance in control rats [81]. Flutamide treatment eliminated the overabundance of Complexes I and II in the former study [78]. The only human study reported no significant difference in pyruvate dehydrogenase (PDH) expression in the control and PCOS uterus samples [76]. No identified studies measured cellular bioenergetics or complex activity. 

Only one study reported mitochondria dynamics in the PCOS-exposed uterus. They found that DHT + insulin-exposed pregnant rats had decreased *Drp1* expression, whereas *Mfn1* and *Opa1* expression was no different from the controls [79]. Because fission is primarily regulated by the post-translational modification (phosphorylation) of DRP1, the significance of this finding in the uterus is unknown.

Like the ovary, DHEA-treated mouse models of PCOS demonstrate more oxidative stress in the uterus. Specifically, they have higher levels of 4-HNE, alongside increased SOD2, which likely increases in response to higher levels of ROS [77]. In these models, L-carnitine and acetyl-L-carnitine returned 4-HNE levels to those of the controls, and propionyl-L-carnitine reduced the abundance of SOD2 [77]. Interestingly, contrasting results were found in pregnant rats exposed to both DHT and insulin. Their uteruses had fewer ROS and less phosphorylated SOD1, GPX4, and glutathione [79,80]. Overall, we surmise that the conflicting biogenesis and ROS outcomes in DHEA-treated mouse models compared to DHT plus insulin-treated gravid rat models may be attributed to differences among species, pregnancy, insulin treatment, the length and type of androgen exposure, as well as the variation in the method of measuring ROS and oxidative regulators. For these reasons, no strong conclusions can be drawn from the compiled studies of mitochondria in the PCOS-exposed uterus.

**Table 4:** Effects of PCOS on mitochondrial function in the uterus.

Mitochondrial Function	Effect of PCOS on Mitochondrial Function	Model/Species	PCOS Model/Diagnosis	Treatment Timeframe	Method	References	Therapeutic Intervention	References
**Biogenesis**	Increased PGC-1α	Mouse	SQ DHEA	20 days	WB	[77]		
	No change in PGC-1α	Rat	IP DHT + INS	GD 0.5–GD 13.5	qPCR	[78]		
	Decreased PGC-1α	Rat	IP DHT + INS	GD 7.5–GD 13.5	qPCR	[79]		
	Increased TFAM	Human	Rotterdam Criteria		WB	[76]		
	No change in TFAM	Rat	IP DHT + INS	GD 7.5–GD 13.5 or GD 14.5	qPCR	[78,79]		
	Decreased NRF1	Rat	IP DHT + INS	GD 7.5–GD 13.5	qPCR	[79]		
**Mitochondrial Genome**	Decreased mtDNA copy number	Rat	IP DHT + INS	GD 7.5–GD 13.5	qPCR	[79]		
**Ultrastructure**	Decreased TOMM20	Mouse	SQ DHEA	20 days	IHC	[77]	L-carnitine/acetyl-L-carnitine returned levels closer to control	[77]
	Increased prohibitin I	Rat	IP DHT + INS	GD 7.5–GD 14.5	WB	[81]		
	Shrunken mitochondria	Rat	IP DHT + INS	GD 7.5–GD 13.5	TEM	[82]		
	Swollen mitochondria	Rat	IP DHT + INS	GD 7.5–GD 13.5 or GD 14.5	TEM	[78,81]	N-acetyl-cysteine improved but did not fully rescue morphology and also impaired mitochondria in controls; flutamide decreased number of small swollen mitochondria but cristae remained disorganized	[78,81]
	Electron-dense and collapsed cristae	Rat	IP DHT + INS	GD 7.5–GD 13.5 or GD 14.5	TEM	[78,81,82]		
**Metabolism**	No difference in VDAC	Rat	IP DHT + INS	GD 7.5–GD 14.5	WB	[81]	N-acetyl-cysteine did not change VDAC but did decrease it in controls	[81]
	Decreased Complex I	Rat	IP DHT + INS	GD 7.5–GD 14.5	WB	[81]	N-acetyl-cysteine normalized	[81]
	Increased Complex I	Rat	IP DHT + INS	GD 0.5–GD 13.5	WB	[78]	Flutamide normalized	[78]
	Increased Complex II	Rat	IP DHT + INS	GD 0.5–GD 13.5	WB	[78]	Flutamide normalized	[78]
	Decreased Complex III	Rat	IP DHT + INS	GD 7.5–GD 13.5	WB	[79,81]	N-acetyl-cysteine normalized	[81]
	No difference in PDH	Human	Rotterdam Criteria		WB	[76]		
**Dynamics**	Decreased DRP1 (Fission)	Rat	IP DHT + INS	GD 7.5–GD 13.5	qPCR	[79]		
	No change in MFN1 (Fusion)	Rat	IP DHT + INS	GD 7.5–GD 13.5	qPCR	[79]		
	No change in OPA1 (Fusion)	Rat	IP DHT + INS	GD 7.5–GD 13.5	qPCR	[79]		
**ROS and Repair**	Increased 4-HNE adducts	Mouse	SQ DHEA	20 days	IHC	[77]	L-carnitine/acetyl-L-carnitine returned levels closer to control	[77]
	Reduced ROS levels	Rat	IP DHT + INS	GD 7.5–GD 13.5	OxiSelect In Vitro ROS/RNS assay	[79]		
	Reduced GPX4	Rat	IP DHT + INS	GD 7.5–GD 13.5	WB, IHC	[82]		
	Reduced glutathione	Rat	IP DHT + INS	GD 7.5–GD 13.5	Glutathione/glutathione + glutathione disulfide assay	[82]		
	Reduced phosphorylated SOD1	Rat	IP DHT + INS	GD 7.5–GD 13.5	WB	[79]		
	Increased SOD2	Mouse	SQ DHEA	20 days	WB	[77]	Propionyl-L-carnitine altered levels	[77]

PGC1α: peroxisome proliferator-activated receptor gamma coactivator 1-alpha; TFAM: mitochondrial transcription factor A; NRF1: nuclear respiratory factor 1; TOMM20: translocase of the outer membrane; VDAC: voltage-dependent anion channel; PDH: pyruvate dehydrogenase; DRP1: dynamin-related protein 1; MFN1: mitofusin 1; OPA1: optic atrophy 1 mitochondrial dynamin-like GTPase; 4-HNE: 4-hydroxynonenal; GPX4: glutathione peroxidase 4; SOD: superoxide dismutase; SQ: subcutaneous; IP: intraperitoneal; DHEA: dehydroepiandosterone; DHT: dihydrotestosterone; INS: insulin; GD: gestational day; WB: Western blot; IHC: immunohistochemistry; TEM: transmission electron microscopy.

## 7. Placenta Mitochondria in PCOS

It is important to consider the influence of PCOS on the placenta, as it relates to pregnancy and fetal health. The placenta’s role in the growth and development of the fetus and its programming of health and disease risk cannot be underestimated [83]. Mitochondria are crucial for nutrient uptake and transport across the placenta and to the fetus. Many PCOS studies focused on hormonal and inflammatory imbalances in the placenta [84,85,86,87,88,89]; however, few studies, especially in humans, focused on mitochondria. This may be due to limited research on PCOS in pregnancy. Nonetheless, we summarize the role of placental mitochondria in influencing outcomes of PCOS pregnancies in Table 5.

Only one study measured markers of mitochondrial biogenesis. They found that placental *Pgc1a*, *Tfam*, and *Nrf1* mRNA expression levels were lower in pregnant rats exposed to DHT and insulin. This was associated with a lower mtDNA copy number, a result that did not occur when rats were exposed to only DHT or only insulin [80]. 

Studies investigating mitochondrial ultrastructure showed that DHT and insulin impact mitochondria in the trophoblasts of the junctional zone but not the labyrinth zone [80,81]. Ultrastructural changes reported included blebbing and a loss of tubular cristae [80,81,82]. Disrupted membranes [81], decreased mitochondria count [80], and increased space between cristae with decreased electron-dense material [80] were also reported. Contrary to its effects on the uterus, high-dose NAC abolished ultrastructural changes in the junctional trophoblasts. However, NAC had no impact on mitochondria in the labyrinth [81].

Only one study measured metabolic function in PCOS placentas. Pregnant rats exposed to DHT and insulin had lower levels of VDAC [81], but there was no difference in Complex III expression by Western blot. Treatment with high-dose NAC improved VDAC levels in affected rats [81]. 

Only one PCOS placental study measured markers of fusion and fission. DHT and insulin-treated pregnant rats had lower *Mfn1* and fission-associated *Drp1*, measured using qPCR [80]. Two studies using DHT and insulin-exposed gravid rat models measured ROS and antioxidant systems. Similar to the ovary and uterus, PCOS increased ROS and lipid peroxidation [80]. There was also increased cytosolic NRF2, and IHC localized NRF2 to the basal zone [80]. Looking at SOD family expression and activity, they found that there was less phosphorylated SOD1 and a lower p-SOD1:SOD1 ratio while there was no change in SOD2 [80]. GPX4 expression was also lower in the junctional and labyrinth zones; however, no differences were reported in whole-tissue GPX4 or glutathione [82].

## 8. Peripheral Markers of Mitochondrial Dysfunction in PCOS

Many studies of PCOS utilized rodent models to investigate the organ-specific impacts and mitochondrial mechanisms of the disease; however, a growing body of research has investigated markers of mitochondrial function in humans using blood and plasma. These studies are summarized in Table 6. Identifying peripheral blood markers associated with mitochondrial quality and quantity offers a translatable and clinically relevant tool to gain insights into the diagnosis, prognosis, and pathophysiology of PCOS. Although more studies are utilizing blood and plasma to identify perturbations in the nuclear and mitochondrial genome, how they cause disease and guide potential therapy remains understudied. 

To date, only one study investigated genetic variations in the drivers of mitochondrial biogenesis [90]. Reddy et al. identified a polymorphism of the *Pgc-1α* gene that might confer a higher risk of developing PCOS. Specifically, 118 South Indian PCOS patients had a higher frequency of an “A” allele in the rs8192678 PGC-1α Gly482Ser polymorphism, and carriers of the “AA” allele had a lower mtDNA copy number [90]. This finding aligns with other studies discussed below. In contrast, a previously reported rs1937 single nucleotide polymorphism (SNP) in the gene that encodes a missense mutation in *Tfam* was not found to be different in PCOS [90].

Studies reporting the mtDNA copy number in the peripheral blood of PCOS patients compared to controls had mixed results. Only one study reported a higher mtDNA copy number [91], whereas multiple studies, including a robust meta-analysis, reported a significantly lower mtDNA copy number [49,92,93,94]. The former study used a multiplex assay that paired the mtDNA copy number with the common mtDNA 4977 bp (mtDNA^4977^) deletion, and the copy number was calculated using a minor arc segment [91]. The investigators also adjusted findings for BMI and hormone levels, which negated the significance [91]. Stronger conclusions were found in a contrasting meta-analysis by Moosa et al., which more convincingly demonstrated a lower circulating mtDNA copy number in women with PCOS (n = 267 vs. 262 controls) [49]. Moreover, this finding is further supported by the animal and tissue studies discussed previously. 

Several studies reported inverse correlations between the mtDNA copy number and insulin resistance, waist circumference, and triglyceride levels, and a positive correlation with sex hormone-binding globulin levels [49,94]. Conversely, Yang et. al. [95] investigated associations between the mtDNA copy number with anthropometric measures and 8-OHdG in PCOS patients. They did not find any correlation between the mtDNA copy number and anthropometric measures at baseline but reported a decreased mtDNA copy number at 6 and 12 months after metformin treatment [95]. Overall, these findings suggest that the mitochondrial copy number in peripheral blood is a useful tool for identifying and tracking co-morbidities in patients with PCOS.

Peripheral blood was also used to identify SNPs within mtDNA that were associated with PCOS. One study found that PCOS was associated with a higher deletion rate of mtDNA^4977^, the most common deletion of the mitochondrial genome [91]. Others found that PCOS was associated with up to a threefold increase in a 9 bp deletion in the mitochondrial genome [49,96,97,98,99]. Specifically, unique variants T12811C and T12338C in the *Nd5* gene [97,100,101], and G8584A and C8684T in the *A6* genes [97,98,100], seem to be specific to PCOS cases and did not appear in control patients. The coding regions for mitochondrial tRNAs in PCOS patients also had variants in the tRNA^Cys^ and tRNA^Leu^ genes appearing more frequently than in control patients, as well as unique variants in the tRNA^Glu^, tRNA^Gln^, tRNA^Lys^, tRNA^Arg^, and tRNA^Asp^ genes that were not present in control patients [49,92,97,100]. Others showed that mtDNA mutations, especially in the D-loop loci, which regulate replication, may be pathogenic and influence co-morbidities such as BMI and insulin resistance [102]. A meta-analysis conducted by Moosa et. al. compiled the polymorphisms present in the noncoding D-Loop and determined that PCOS patients had lower odds than the controls of having the C150T and T146C polymorphisms and about the same odds of having the A263G polymorphism [49]. In a South Indian cohort, one of these studies sequencing the D-loop of the mitochondrial genome found two significantly different SNPs in PCOS (A189G and D310) [93]. The carriers of these SNPs additionally had significantly lower mtDNA copy numbers [93]. The presence of multiple SNPs at various coding and non-coding regions of mtDNA in peripheral blood warrants the investigation of the impacts of these mutations and downstream mechanisms in the pathophysiology of PCOS. 

We found no peripheral blood studies that investigated mitochondrial ultrastructure, metabolism, or dynamics. Only one study utilized human plasma to assess the systemic response to reactive oxygen species in PCOS patients. Peroxiredoxin 3 (PRX3), an antioxidant enzyme, was measured alongside glucose and insulin levels following an oral glucose tolerance test (OGTT). As expected, PCOS patients had consistently higher levels of glucose and insulin, and a positive correlation was found between insulin at one hour and PRX3 at two hours in PCOS cases. This suggests that insulin surges are followed by oxidative stress in PCOS patients [103]. 

Although studies investigating peripheral blood markers of mitochondrial dysfunction in PCOS can leverage human patients, more work is necessary to understand how these correlate with the mechanisms of the disease. Nevertheless, this crucial work can help identify polymorphisms associated with PCOS that might be used as biomarkers accessible by minimally invasive techniques. Additionally, studies that assess mitochondrial dysfunction peripherally aid in our understanding of the systemic effects of not only PCOS but also other metabolic and endocrine disorders that impact the entire body. 

**Table 6:** Effects of PCOS on mitochondrial function in the peripheral blood and plasma of humans diagnosed by Rotterdam criteria.

Mitochondrial Function	Effect of PCOS on Mitochondrial Function	Method	Therapeutic Intervention	References
**Biogenesis**	Reduced “GG”(WT) frequency of PGC-1α rs8192678 polymorphism	PCR, RFLP Analysis		[90]
	No difference in TFAM genotype or allele frequency	PCR, RFLP Analysis		[90]
**Mitochondrial Genome**	Lower mtDNA copy number	qPCR		[92,93,94]
	Higher mtDNA copy number	qPCR		[91]
	Negative association between mtDNA copy number and fasting insulin, HOMA-IR, waist circumference, and triglycerides	Pearson correlation coefficient		[94]
	Positive association between mtDNA copy number and quantitative insulin-sensitivity check index (QUICKI) and sex hormone-binding globulin (SHBG)	Pearson correlation coefficient		[94]
	No correlation between mtDNA copy number and anthropometric measure or 8-OHdG	qPCR, 8OH-dG ELISA kit	Metformin decreased mtDNA copy number at 6 and 12 months of treatment	[95]
	Higher mtDNA^4977^ deletion rate	qPCR		[91]
	Higher frequency of a 9 bp deletion	qPCR		[49,96,97,98,99]
	ND5 gene polymorphisms: T12811C, T12338C	qPCR		[97,100,101]
	A6 gene polymorphisms: G8584A, C8684T	qPCR		[97,98,100]
	Unique tRNA variants and higher frequency of variants for Cys and Leu tRNAs	qPCR		[92,97,100]
	Greater frequency of D-loop SNPs C150T, T146C, A189G, and D310	PCR, Mitomap, and mtDB mitochondria databases		[49,93]
	Carriers of AA genotype of PGC1a polymorphism rs8192678 and D-loop SNPs A189G and D310 had lower mtDNA	PCR, Mitomap, and mtDB mitochondria databases		[90,93]
**Ultrastructure**	No Reports			
**Metabolism**	No Reports			
**Dynamics**	No Reports			
**ROS and Repair**	Decreased PRX3 2–3 h post-OGTT	ELISA		[103]
	Positive correlation between PRX3 at 2 h post-OGTT and insulin at 1 h post-OGTT	Spearman correlation analysis		[103]

PGC1α: peroxisome proliferator-activated receptor gamma coactivator 1-alpha; TFAM: mitochondrial transcription factor A; HOMA-IR: Homeostatic Model Assessment for Insulin Resistance; PRX3: peroxiredoxin 3; OGTT: oral glucose tolerance test; RFLP: restriction fragment length polymorphism.

## 9. Conclusions

Across reproductive organs, many studies have demonstrated PCOS-induced changes in mitochondrial health. The summarized evidence suggests that PCOS alters both mitochondrial quantity and quality, which are likely to contribute to both reproductive and transgenerational consequences. This in-depth review provides insights into the role of mitochondria in PCOS pathophysiology and lays the foundation of knowledge needed to develop diagnostic, intervention, and prevention strategies that will improve reproductive and metabolic health for people with PCOS and their progeny. 

Although this review was quite extensive, there is much more to consider about the role of mitochondria in the pathogenesis of PCOS. For example, many studies highlighted by our methods showed impaired mitochondrial dynamics, metabolism, and ROS production in the ovary, uterus, and placenta of PCOS patients; however, it is also important to consider how this disruption affects mitochondrial interactions with other organelles, namely the endoplasmic reticulum, lysosomes, and nucleus, in order to better understand the intracellular networks underlying PCOS pathophysiology. Another limitation is that this review focused primarily on the reproductive system, but it is likely that PCOS affects mitochondria in skeletal muscle, liver, pancreas, kidney, thyroid, adipose tissue, and leukocytes, thereby impacting the metabolic health of both parent and offspring across the lifespan. Given the extent of mitochondrial dysfunction highlighted in this review, mitochondria-targeted therapies would almost certainly improve reproductive and systemic outcomes in PCOS. Treatments evaluated in reproductive organs in this review included metformin and supplemental antioxidants like selenium and cangfudaotan. However, there are certainly many other therapeutic considerations such as MitoQ10, a mitochondrial-targeted coenzyme Q10 antioxidant [104], and myo-inositol, which has been used to improve in vitro fertilization rates and insulin resistance [105]. Overall, this compilation shows that both mitochondrial quantity and quality play a significant role in the pathophysiology of PCOS, and there is a great opportunity to develop mitochondria-targeted therapies that could decrease the reproductive and systemic burdens of this complex and prevalent disease.

## Figures and Tables

**Table 5:** Effects of PCOS on mitochondrial function in the gravid rat placenta with IP DHT + INS injections.

Mitochondrial Function	Effect of PCOS Condition on Mitochondrial Function	Treatment Timeframe	Method	Therapeutic Intervention	References
**Biogenesis**	Decreased PGC-1α	GD 7.5–GD 13.5	qPCR		[80]
	Decreased TFAM	GD 7.5–GD 13.5	qPCR		[80]
	Decreased NRF1	GD 7.5–GD 13.5	qPCR		[80]
**Mitochondrial Genome**	Decreased mtDNA copy number	GD 7.5–GD 13.5	qPCR		[80]
**Ultrastructure**	Mitochondrial blebbing	GD 7.5–GD 13.5 and GD 14.5	TEM		[80,81,82]
	Lack of tubular cristae	GD 7.5–GD 13.5 or GD 14.5	TEM		[81,82]
	Decreased intracristal dilatation	GD 7.5–GD 13.5	TEM		[80]
	Morphology changes limited to junctional zone; little change in the labyrinth zone	GD 7.5–GD 13.5 or GD 14.5	TEM	High-dose NAC abolished damaged morphology in junctional zone but had no effect in labyrinth zone	[81,82]
**Metabolism**	No change in Complex III expression	GD 7.5–GD 14.5	WB	Low-dose NAC decreased Complex I abundance	[81]
	Decreased VDAC	GD 7.5–GD 14.5	WB	High-dose NAC increased VDAC abundance	[80,81]
**Dynamics**	Decreased MFN1 (Fusion)	GD 7.5–GD 13.5	qPCR		[80]
	Decreased DRP1 (Fission)	GD 7.5–GD 13.5	qPCR		[80]
**ROS and Repair**	Increased ROS	GD 7.5–GD 13.5	OxiSelect In Vitro, ROS/RNS assay		[80]
	Increased MDA	GD 7.5–GD 13.5	MDA ELISA		[80]
	Increased cytosolic NRF2 and decreased nuclear NRF2 in basal zone	GD 7.5–GD 13.5	WB, IHC		[80]
	Reduced phosphorylated SOD1 and p-SOD1:SOD1 ratio	GD 7.5–GD 13.5	WB		[80]
	No difference in SOD2 abundance	GD 7.5–GD 13.5	WB		[80]
	No difference in GPX4 abundance	GD 7.5–GD 13.5	WB		[82]
	Less GPX4 in junctional and labyrinth zones	GD 7.5–GD 13.5	IHC		[82]
	No GPX4 in nuclei of spongiotrophoblasts, cytotrophoblasts, and synctiotrophoblasts	GD 7.5–GD 13.5	IHC		[82]
	No difference in glutathione	GD 7.5–GD 13.5	Glutathione/glutathione + glutathione disulfide assay		[82]

PGC1α: peroxisome proliferator-activated receptor gamma coactivator 1-alpha; TFAM: mitochondrial transcription factor A; NRF1: nuclear respiratory factor 1; VDAC: voltage-dependent anion channel; MFN1: mitofusin 1; DRP1: dynamin-related protein 1; MDA: malondialdehyde; NRF2: nuclear respiratory factor 2; SOD: superoxide dismutase; GPX4: glutathione peroxidase 4; IP: intraperitoneal; DHT: dihydrotestosterone; INS: insulin; GD: gestational day; WB: Western blot; IHC: immunohistochemistry; TEM: transmission electron microscopy; NAC: n-acetyl-cysteine.

## Data Availability

Not applicable.
